# Early Mycobacterial Antigens in the Immunodiagnosis of Latent Tuberculosis Infection

**DOI:** 10.3390/pathogens15020181

**Published:** 2026-02-06

**Authors:** Aigul Utegenova, Lazzat Kassayeva, Bayan Turdalina, Aliya Baiduissenova, Ayaz Yktiyarov, Marat Dusmagambetov, Evgeni Sokurenko

**Affiliations:** 1 Department of Microbiology and Virology, Astana Medical University, Astana 010000, Kazakhstan; utegenova.a@amu.kz (A.U.); baiduissenova.a@amu.kz (A.B.); yktiyarov.a@amu.kz (A.Y.); dusmagambetov.m@amu.kz (M.D.); 2 Department of Phthisiopulmonology and Radiology, South Kazakhstan Medical Academy, Shymkent 160019, Kazakhstan; 3 Department of Pediatric Infectious Diseases, Astana Medical University, Astana 010000, Kazakhstan; 4 Department of Microbiology, School of Medicine, University of Washington, Seattle, WA 98195, USA; evs@uw.edu

**Keywords:** latent tuberculosis infection, ESAT-6, CFP-10, QuantiFERON-TB Gold Plus, T-SPOT.TB, interferon-gamma release assay, IP-10, DosR antigens

## Abstract

Latent tuberculosis infection (LTBI) represents a major global health concern as it constitutes the principal reservoir for future tuberculosis (TB) disease. Its identification is particularly important in Bacille Calmette–Guérin (BCG)-vaccinated populations, where cross-reactivity of purified protein derivative limits the specificity of the tuberculin skin test and hampers targeted preventive therapy. Early *Mycobacterium tuberculosis* antigens encoded within the RD1 region, especially ESAT-6, CFP-10 and TB7.7, have enabled the development of antigen-specific interferon-gamma release assays (IGRAs) and recombinant skin tests with improved BCG-independent specificity. This narrative review integrates and critically appraises current evidence on the immunobiological properties of early and latency-associated antigens, the cellular mechanisms underlying T-cell-dependent immune reactivity, and the diagnostic performance of IGRAs and ESAT-6/CFP-10-based skin tests, rather than merely summarizing individual studies. Although these platforms rely on different assay principles (in vitro cytokine release versus in vivo delayed-type hypersensitivity), both measure antigen-specific T-cell memory and do not define the biological stage of infection or reliably distinguish latent from incipient or active TB. Across most adult populations, IGRAs demonstrate high specificity and acceptable sensitivity, whereas reduced sensitivity and higher rates of indeterminate results are observed in young children and immunocompromised individuals. ESAT-6/CFP-10-based skin tests show diagnostic accuracy comparable to IGRAs and may offer operational advantages in resource-limited settings. Latency-associated antigens and host biomarkers such as IP-10, together with multi-analyte immune signatures, represent promising avenues for improving diagnostic sensitivity and prognostic stratification but currently lack sufficient validation for routine clinical use. Overall, RD1-encoded antigens remain central to LTBI immunodiagnosis, while future research should focus on developing stage-resolving and prognostic biomarkers, optimized antigen panels, and standardized interpretive frameworks.

## 1. Introduction

Tuberculosis (TB) remains one of the leading infectious causes of death worldwide despite major advances in diagnosis, prevention, and treatment. According to the most recent World Health Organization (WHO) Global Tuberculosis Report, approximately one quarter of the global population is infected with *Mycobacterium tuberculosis* (*M. tuberculosis*), the majority of whom harbor latent tuberculosis infection (LTBI), defined as a state of persistent immune sensitization in the absence of clinical or microbiological evidence of active disease (WHO 2024) [1,2,3,4]. Individuals with LTBI constitute the main reservoir for future TB cases, with a lifetime risk of reactivation of approximately 5–10%, which is substantially higher among persons with immunosuppression, HIV infection, or other clinical risk factors [2,5,6,7]. Consequently, accurate identification of LTBI and, critically, the ability to distinguish latent infection from early active disease and to predict progression remain central challenges for global TB elimination strategies and are key priorities in current WHO policy documents (WHO 2024) [4,5,6,7,8].

For decades, LTBI screening relied almost exclusively on the tuberculin skin test (TST) based on purified protein derivative (PPD). However, PPD contains a heterogeneous mixture of antigens shared with Bacille Calmette–Guérin (BCG) vaccine strains and many nontuberculous mycobacteria, resulting in reduced specificity and frequent false-positive reactions in BCG-vaccinated populations [3,4,7,9]. A major breakthrough was the identification of the Region of Difference-1 (RD1) in the *M. tuberculosis* genome, which is absent from all BCG strains and most environmental mycobacteria and encodes highly immunogenic early secreted antigens, most notably ESAT-6 (early secreted antigenic target-6) and CFP-10 (culture filtrate protein-10) [10,11,12]. These antigens elicit strong T-cell-mediated interferon-γ (IFN-γ) responses and play a central role in host–pathogen interactions during early infection, granuloma formation, and bacterial dissemination [11,12,13,14].

Incorporation of ESAT-6 and CFP-10 into interferon-gamma release assays (IGRAs) and recombinant antigen-based skin tests has substantially improved diagnostic specificity compared with TST, particularly in populations with high BCG coverage [3,7,15,16,17,18,19,20,21]. Nevertheless, these assays detect antigen-specific cellular immune memory rather than bacterial metabolic activity, and a positive result does not define the biological stage of infection. Current IGRAs and ESAT-6/CFP-10-based skin tests therefore cannot reliably discriminate latent infection from incipient or active tuberculosis, nor can they accurately predict which infected individuals will progress to disease [3,6,17,18,19,20,22,23,24]. This limited prognostic performance represents a major unmet clinical need and is explicitly emphasized in the WHO Target Product Profiles for novel TB diagnostics, which call for tests capable of identifying individuals at highest risk of progression from infection to active disease.

Beyond RD1 antigens, increasing attention has focused on latency-associated proteins, particularly those regulated by the DosR dormancy regulon and resuscitation-promoting factors, which are preferentially expressed during non-replicating persistence and may better reflect immunological states associated with LTBI [25,26,27,28,29,30,31]. Panels combining early secreted RD1 antigens with latency-associated targets, together with alternative immune readouts such as IP-10, are being explored to enhance diagnostic sensitivity and to improve risk stratification for progression from latent infection to active disease, although their clinical validation and standardization remain incomplete [29,30,31,32,33].

Given both the advances achieved with RD1-based diagnostics and their persistent limitations, a concise synthesis of the immunobiological properties of early and latency-associated *M. tuberculosis* antigens, their performance in contemporary diagnostic platforms, and their potential role in prognostic assessment is warranted. Therefore, this review summarizes current evidence on ESAT-6, CFP-10, TB7.7, and selected latency-associated antigens, evaluates the diagnostic accuracy and clinical utility of IGRAs and ESAT-6/CFP-10-based skin tests in comparison with TST, and discusses emerging biomarker strategies aimed at improving identification of individuals with LTBI who are at highest risk of progression to active tuberculosis [1,2,3,4,5,6,7,8,14,15,16,17,18,19,20,21,23,24,25,26,27,28,29,30,31,32,33].

## 2. Materials and Methods

### 2.1. Study Design

**Figure 1:**
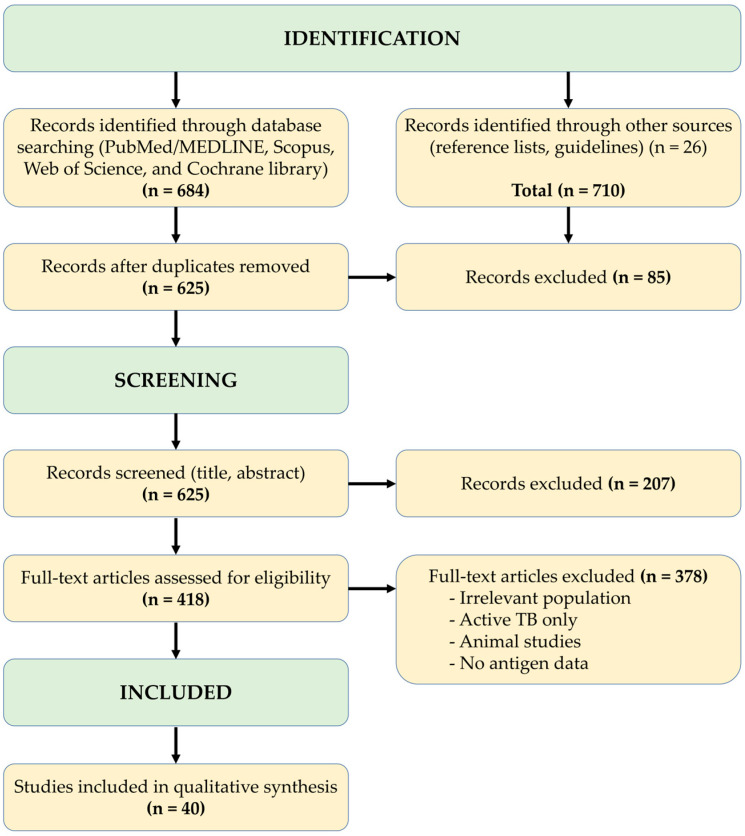
PRISMA-style flow diagram of the literature search and study selection.

This work was conducted as a narrative literature review with structured elements of evidence synthesis, focusing on early *Mycobacterium tuberculosis* antigens (ESAT-6, CFP-10, TB7.7), selected latency-associated antigens regulated by the DosR dormancy regulon and resuscitation-promoting factors, and their application in the immunodiagnosis and risk stratification of latent tuberculosis infection (LTBI). No formal systematic review protocol (e.g., PROSPERO) was registered and no quantitative meta-analysis was performed. A PRISMA 2020-style flow diagram was used to transparently report the processes of literature identification, screening, eligibility assessment, and final study inclusion (Figure 1).

### 2.2. Search Strategy

A comprehensive literature search was performed in PubMed/MEDLINE, Scopus, Web of Science, and the Cochrane Library to identify peer-reviewed publications from January 2015 to November 2025. The following keywords and Boolean combinations were used: “latent tuberculosis infection”, “ESAT-6”, “CFP-10”, “RD1 antigens”, “interferon-gamma release assay”, “QuantiFERON-TB Gold Plus”, “T-SPOT.TB”, “TB7.7”, “DosR dormancy regulon”, “resuscitation-promoting factors”, “ESAT-6 CFP-10 skin test”, “C-Tb test”, “IP-10 biomarker”, and “diagnostic performance tuberculosis”.

Additional records were identified by manual screening of reference lists of key reviews, international guidelines, and consensus documents.

In total, 684 records were retrieved from electronic databases and 26 additional records from other sources (reference lists and guidelines), yielding 710 records overall (Figure 1).

### 2.3. Study Selection

After removal of duplicates, 625 unique records remained. Titles and abstracts of these records were screened for relevance to LTBI immunodiagnosis using RD1-encoded antigens and latency-associated biomarkers. During this phase, 207 records were excluded because of irrelevant topic, exclusive focus on active tuberculosis, non-human studies, or absence of antigen-based diagnostic data.

A total of 418 full-text articles were subsequently assessed for eligibility. Of these, 378 were excluded for one or more of the following reasons:

(i) inappropriate study population;
(ii) exclusive investigation of active TB without LTBI relevance;
(iii) animal or in-vitro studies without translational applicability;
(iv) lack of data on ESAT-6, CFP-10, TB7.7, or latency-associated antigens;
(v) insufficient methodological detail.

Finally, 40 studies fulfilled all inclusion criteria and were incorporated into the qualitative synthesis (Figure 1).

### 2.4. Eligibility Criteria

Studies were included if they met all of the following criteria:

Study type: original clinical, observational, diagnostic-accuracy, or longitudinal studies; systematic reviews; meta-analyses; or international guidelines.

Population: human subjects undergoing LTBI screening or TB infection risk assessment.

Index tests: IGRAs or skin tests based on ESAT-6, CFP-10, TB7.7, DosR-regulated antigens, or resuscitation-promoting factors.

Outcomes: diagnostic performance, immune response profiles, or prognostic value for progression to active TB.

Language and period: English-language publications from 2015 onward.

### 2.5. Data Extraction and Synthesis

From each eligible study, the following data were extracted: author, year of publication, study design, sample size, study population, antigen(s) evaluated, diagnostic platform (e.g., QuantiFERON-TB Gold Plus, T-SPOT.TB, ESAT-6/CFP-10 skin tests), main outcome measures, and key findings.

Because of heterogeneity in study design, antigen panels, and outcome definitions, a quantitative meta-analysis was not attempted. Instead, results were synthesized narratively and structured into thematic domains:

(i) immunobiological properties of RD1 and latency-associated antigens;
(ii) diagnostic performance of ESAT-6/CFP-10-based IGRAs and skin tests;
(iii) performance in special populations (children, healthcare workers, immunocompromised individuals);
(iv) emerging biomarker panels (IP-10, multi-cytokine signatures);
(v) prognostic implications and alignment with WHO Target Product Profiles.

### 2.6. Ethical Considerations

This study was based exclusively on analysis of published literature and did not involve primary data collection from human participants. Therefore, institutional ethical approval was not required.

## 3. Results

### 3.1. Immunological Characteristics of Early Mycobacterium tuberculosis Antigens

Genomic and comparative analyses have identified the Region of Difference 1 (RD1) as a key locus present in virulent members of the *Mycobacterium tuberculosis* complex but absent from all Bacille Calmette–Guérin (BCG) vaccine strains and most nontuberculous mycobacteria [10,11,12]. RD1 encodes several early secreted virulence factors, among which ESAT-6 (Early Secreted Antigenic Target-6) and CFP-10 (Culture Filtrate Protein-10) are the most extensively characterized and immunodominant [10,11,12,13,14]. These proteins are secreted via the ESX-1 type VII secretion system, form a stable heterodimeric complex, and contribute to host–cell membrane perturbation, granuloma dynamics, and bacterial dissemination during early stages of infection [11,12,13,14].

Immunologically, ESAT-6 and CFP-10 are recognized early after infection by both CD4^+^ and CD8^+^ T lymphocytes, inducing a predominantly Th1-polarized response characterized by production of interferon-gamma (IFN-γ), tumor necrosis factor-α (TNF-α), and interleukin-2 (IL-2) [11,12,13,14,15]. Epitope-mapping studies have demonstrated multiple HLA class II-restricted epitopes within both antigens, explaining their broad population coverage and consistent immunogenicity across diverse genetic backgrounds [11,12,13]. In addition, enrichment of ESAT-6-specific CD8^+^ T-cell responses has been reported in individuals with recent infection and incipient tuberculosis, providing a biological rationale for the incorporation of CD8-targeting peptide pools into newer interferon-gamma release assay (IGRA) formats [16,18,19,20].

TB7.7 (Rv2654c), another RD1-associated antigen, has been included in some commercial IGRA platforms as an additional early secreted target. Clinical studies, particularly in high-burden settings and among people living with HIV, suggest that inclusion of TB7.7 may modestly increase assay sensitivity without a substantial loss of specificity, although results remain heterogeneous and this antigen is not universally implemented across diagnostic systems [16,17,18].

Beyond RD1-encoded antigens, increasing attention has focused on latency-associated proteins, especially those regulated by the DosR dormancy regulon (e.g., Rv1733c, Rv2626c, Rv2628, Rv2004c) and the family of resuscitation-promoting factors (Rpf). These antigens are preferentially expressed under hypoxic and stress conditions associated with non-replicating persistence and are thought to reflect immunological states characteristic of latent infection [27,28,29,30,31]. Several studies have demonstrated stronger or more frequent T-cell responses to DosR-regulated antigens in individuals with LTBI compared with patients with active disease, supporting their potential utility as complementary immunodiagnostic markers and as components of extended antigen panels aimed at improving stage discrimination and risk stratification [34,35,36].

The principal immunobiological features, functional roles, and diagnostic relevance of RD1-encoded and latency-associated antigens are summarized in Table 1.

### 3.2. Diagnostic Performance of ESAT-6/CFP-10-Based Interferon-Gamma Release Assays

Interferon-gamma release assays (IGRAs) incorporating the RD1-encoded antigens ESAT-6 and CFP-10 represent the most widely used antigen-specific tools for the immunodiagnosis of *Mycobacterium tuberculosis* infection. Their diagnostic performance has been evaluated in diverse populations, including adults, children, healthcare workers, and immunocompromised individuals, with respect to sensitivity, specificity, rate of indeterminate results, and ability to reflect recent exposure. A structured summary of the main performance characteristics of ESAT-6/CFP-10-based IGRAs across different clinical settings is provided in Table 2.

#### 3.2.1. Adult General and High-Risk Populations

Multiple clinical studies and diagnostic-accuracy evaluations of QuantiFERON-TB Gold Plus (QFT-Plus) and T-SPOT.TB indicate that ESAT-6/CFP-10-based IGRAs have high specificity for *M. tuberculosis* infection and provide clinically useful evidence of immune sensitization in the absence of active disease [15,16,17,18,19,20,21]. Importantly, IGRAs do not directly establish the biological “stage” of infection; rather, they support the operational diagnosis of LTBI when results are interpreted together with clinical evaluation and the exclusion of active tuberculosis [3,6,17,18,19,20]. In adult cohorts, IGRA sensitivity is most commonly estimated using microbiologically confirmed active TB as a reference condition for infection status, with values typically ranging from ~80–90%, while specificity in low-risk, BCG-vaccinated populations frequently exceed 95% [15,16,17,18,19].

Comparative analyses of QFT-Plus versus the earlier QFT-GIT generally show non-inferior or slightly higher sensitivity with preserved specificity, which is an effect attributed mainly to the separate TB1 (predominantly CD4-driven) and TB2 (combined CD4/CD8-driven) antigen tubes [15,16,17,18]. Several prospective contact-tracing studies report that TB2 responses, reflecting CD8-directed stimulation, are more frequently positive among recent contacts and individuals with radiological or clinical indicators suggestive of incipient disease, supporting the hypothesis that CD8 responses may be enriched in recent or higher-intensity antigen exposure [16,18,19,20].

The diagnostic performance of T-SPOT.TB, which enumerates ESAT-6- and CFP-10-responsive IFN-γ-secreting cells, is broadly comparable to QFT-Plus in head-to-head evaluations [15,16,17,21]. Some evidence suggests that T-SPOT.TB may yield fewer indeterminate results in individuals with severe lymphopenia due to standardized peripheral blood mononuclear cell input; however, it is technically more demanding and resource-intensive [17,21].

#### 3.2.2. Healthcare Workers and Serial-Screening Cohorts

Among healthcare workers and other occupational groups undergoing serial screening, interferon-gamma release assays based on RD1-encoded *Mycobacterium tuberculosis* antigens (ESAT-6 and CFP-10) identify a higher baseline prevalence of LTBI than the tuberculin skin test; however, conversion and reversion phenomena are common [18,19,34]. Apparent conversions to ESAT-6/CFP-10 often occur near the assay cut-off, and reversions are frequently observed on repeat testing, raising questions about the relative contributions of true changes in antigen-specific T-cell reactivity versus analytical and biological variability [18,19]. Quantitative analyses indicate that higher baseline IFN-γ responses to these antigens and larger increases over time are more strongly associated with documented exposure and recognized risk factors, whereas small “borderline” fluctuations around the cut-off may reflect non-specific variability rather than stable *M. tuberculosis* infection [18,19,34]. These findings have led several authors to propose borderline or uncertainty zones for ESAT-6/CFP-10-based IGRAs and to recommend confirmatory repeat testing in cases of minor quantitative changes [18,34].

#### 3.2.3. Children and Adolescents

Performance of ESAT-6/CFP-10-based IGRAs in the diagnosis of latent tuberculosis infection in children is more heterogeneous than in adults. Systematic reviews and pediatric cohort studies indicate that, in BCG-vaccinated settings, IGRAs are at least as specific as the tuberculin skin test and often more specific; however, their sensitivity for identifying LTBI appears reduced in very young children (<5 years) [21,22,23,24,25,26,29]. In pediatric contacts of infectious TB cases, QuantiFERON-TB Gold Plus and T-SPOT.TB generally show good concordance, but indeterminate or low-level responses are more frequent in infants and in children with severe malnutrition or HIV infection, likely reflecting developmental immaturity or impairment of antigen-specific T-cell responses to ESAT-6 and CFP-10 [21,22,23,26]. These age-related immunological factors are clinically relevant, as young children with LTBI are at higher risk of progression to active disease, underscoring the need for careful interpretation of IGRA results and, where appropriate, the use of complementary diagnostic approaches such as ESAT-6/CFP-10-based skin tests or integrated clinical risk assessment in this population.

#### 3.2.4. Immunocompromised Populations

In immunocompromised individuals—including people living with HIV, patients with chronic kidney disease or autoimmune disorders, and those receiving immunosuppressive therapy—ESAT-6/CFP-10-based IGRAs remain more specific than the tuberculin skin test, but their sensitivity is reduced and indeterminate results are more frequent due to impaired antigen-specific T-cell function [17,18,19,20,29,34]. In these populations, the risk of progression from latent infection to active tuberculosis is increased, and active disease is often diagnosed by direct microbiological methods such as culture or molecular detection rather than by immunological assays. Nevertheless, after active TB has been excluded, identification of LTBI remains clinically important for guiding preventive therapy. Data from cohorts of adults living with HIV and transplant candidates indicate that combined strategies (IGRA together with TST or ESAT-6/CFP-10-based skin tests) may increase the detection of latent infection, although discordant results are common and complicate interpretation [17,18,19,20,34]. QuantiFERON-TB Gold Plus may offer some advantage over QFT-GIT through inclusion of TB2-tube CD8-associated responses, but available evidence is heterogeneous and not fully consistent across studies [16,17,18,19,20].

### 3.3. Quantitative Responses, Serial Testing, and Risk of Progression

Several longitudinal studies have assessed the association between quantitative IGRA responses to ESAT-6 and CFP-10 and the subsequent risk of progression to active TB. Overall, individuals with higher baseline interferon-gamma (IFN-γ) concentrations or greater spot-forming cell counts appear to have a higher relative risk of developing TB than those with low-level positive results; however, the absolute predictive value remains modest [3,6,30,31,32,34]. Only a small proportion of IGRA-positive individuals progress to active disease within 2–3 years, and a substantial fraction of incident TB cases occur among persons who initially test IGRA-negative [3,6,30,31,32].

In serial testing of close contacts, new IGRA conversion within months of exposure—particularly when accompanied by large quantitative increases in ESAT-6/CFP-10 responses—has been more strongly associated with radiographic abnormalities and indicators of incipient TB [18,19,30]. Nevertheless, no universally accepted quantitative threshold has emerged that reliably stratifies individuals into high- versus low-risk categories. These observations reinforce the need for adjunctive prognostic biomarkers and optimized antigen panels to improve risk prediction beyond the current IGRA platforms [29,30,31,32,33].

### 3.4. ESAT-6/CFP-10-Based Skin Tests

The main diagnostic characteristics of ESAT-6/CFP-10-based recombinant skin tests, including C-Tb, ECT formulations, and Diaskintest, in different populations are summarized in Table 3.

#### 3.4.1. C-Tb Skin Test

The C-Tb skin test uses a standardized intradermal dose of a recombinant ESAT-6/CFP-10 fusion protein. Phase 2 and 3 trials in adults and children have shown that its diagnostic sensitivity for culture-confirmed pulmonary TB is broadly comparable to that of IGRAs (as summarized in Table 3), typically in the range of 75–85% [21,22,23]. Specificity in low-risk, BCG-vaccinated populations generally exceed that of the TST and is frequently reported as >95% when using optimized positivity thresholds. Induration cut-offs of ≥5–8 mm appear to provide the most balanced trade-off between sensitivity and specificity [21,22,23].

Among people living with HIV, C-Tb has demonstrated acceptable sensitivity and high specificity, together with a favorable safety profile that is at least comparable with TST, with most adverse events consisting of mild local reactions [22,23]. Agreement between C-Tb and IGRAs is usually moderate to substantial, whereas concordance with TST tends to be lower, particularly in settings with widespread BCG vaccination [21,22,23].

#### 3.4.2. ESAT-6/CFP-10 (ECT) and Other Recombinant Skin Tests

Several ESAT-6/CFP-10-based skin tests (sometimes collectively referred to as ECT tests) have been evaluated, particularly in China and the Russian Federation [24,25,26,27,28,29]. Data from large randomized and observational studies comparing ECT formulations with TST and IGRAs indicate that these tests consistently demonstrate higher specificity than TST in BCG-vaccinated populations, while maintaining sensitivity for active TB and LTBI that is broadly comparable to IGRAs [24,25,26,27]. Reported diagnostic performance appears relatively stable across age groups, including children, although evidence in infants remains limited [24,25,26,27].

Systematic reviews synthesizing global experience with recombinant ESAT-6/CFP-10-based skin tests, including C-Tb, ECT formulations and Diaskintest, conclude that these assays represent BCG-independent intradermal alternatives with diagnostic accuracy similar to that of IGRAs and superior to TST, while potentially offering greater feasibility and cost-effectiveness in decentralized or resource-limited settings [28,29,30]. Occasional false-positive ECT reactions attributed to exposure to certain nontuberculous mycobacterial species have been reported, although such cross-reactivity appears uncommon in most published series [28,29,30].

### 3.5. Latency-Associated Antigens and Extended Antigen Panels

The main characteristics and diagnostic implications of latency-associated *Mycobacterium tuberculosis* antigens regulated by the DosR dormancy regulon, resuscitation-promoting factors, and host biomarkers induced by ESAT-6/CFP-10 stimulation are summarized in Table 4.

#### 3.5.1. DosR-Regulated Antigens

Multiple studies have evaluated T-cell responses to antigens regulated by the DosR dormancy regulon (e.g., Rv1733c, Rv2626c, Rv2628, Rv2004c) in individuals with LTBI and active TB. Cross-sectional and case–control investigations generally report higher frequencies and magnitudes of responses to DosR-regulated antigens in persons with LTBI compared with those with active TB. These latency antigens also demonstrate partially complementary recognition patterns relative to ESAT-6/CFP-10, with some individuals classified as LTBI showing reactivity to DosR antigens despite weak or absent RD1-specific responses [27,28,29,30].

Several diagnostic evaluations suggest that antigen panels combining ESAT-6/CFP-10 with selected DosR-regulated proteins may enhance discrimination between LTBI and active TB, particularly when multi-parameter immune readouts such as polyfunctional T-cell responses or composite cytokine signatures are applied [27,28,29,30,31]. However, considerable heterogeneity in antigen selection, assay platforms, study design, and outcome definitions limits direct comparability across studies and prevents firm conclusions regarding the optimal antigen combinations for clinical use.

#### 3.5.2. Resuscitation-Promoting Factors (Rpf) and Other Candidates

Resuscitation-promoting factors (Rpfs), which are implicated in the reactivation of dormant bacilli, also elicit measurable T-cell responses in both LTBI and active TB. Several studies indicate that Rpf-specific responses are more evenly distributed across the LTBI and active-disease spectrum than those elicited by DosR-regulated antigens, potentially reflecting immune recognition at different stages of mycobacterial metabolic activity [29,30,31]. When incorporated into extended antigen panels together with DosR-regulated proteins and RD1-encoded antigens, Rpf-based stimulants may add incremental discriminatory information in certain models; however, their standalone diagnostic performance remains limited [29,30,31].

Genome-wide and proteomic antigen-screening approaches have identified additional candidate proteins that appear to be differentially recognized in LTBI versus active TB. Most of these antigens, however, remain in early-stage evaluation and are not yet integrated into routine diagnostic assays [29,30,31,36,37,38].

### 3.6. Biomarkers Induced by Early Antigens and Multi-Analyte Signatures

#### 3.6.1. IP-10 (CXCL10) and Cytokine Readouts

A substantial number of studies have evaluated secretion of interferon-gamma-inducible protein-10 (IP-10, CXCL10) following ESAT-6/CFP-10 stimulation as an alternative or adjunctive biomarker to interferon-gamma (IFN-γ). Across diverse adult and paediatric cohorts, IP-10 has generally demonstrated sensitivity for TB infection that is comparable to, or in some settings higher than, IFN-γ-based readouts, with particularly favourable performance reported among children, people living with HIV, and other immunocompromised populations in whom IFN-γ responses may be attenuated [29,30,31,32,33]. Specificity of IP-10 is typically similar to that of IGRA-based IFN-γ detection when induced by the same ESAT-6/CFP-10 antigens [29,30,31,32,33]. Dual-marker algorithms combining IFN-γ and IP-10 may modestly increase sensitivity, although often at the expense of a small reduction in specificity. From an operational perspective, IP-10 can be quantified using conventional ELISA or multiplex immunoassay platforms and may be adaptable to dried-blood-spot formats, which is potentially advantageous in low-resource or decentralized settings [29,30,31,32,33].

#### 3.6.2. Multi-Cytokine and Machine-Learning Approaches

Several exploratory studies have investigated multi-analyte cytokine panels (e.g., IFN-γ, IP-10, interleukin-2, tumor necrosis factor-alpha, granulocyte–macrophage colony-stimulating factor) and polyfunctional T-cell profiles following stimulation with ESAT-6/CFP-10, often in combination with latency-associated antigens [29,30,31,32,33]. Using unsupervised clustering or machine-learning-based classification, some investigators have reported improved discrimination between LTBI, active TB, and uninfected controls compared with single-analyte IGRA readouts [30,31,32,33]. However, these signatures are generally assay-specific, frequently require sophisticated laboratory and analytical infrastructure, and have not yet been validated in large, multicentre prospective cohorts. As such, their current role in routine clinical LTBI screening remains exploratory.

### 3.7. Summary of Evidence

Taken together, the available evidence indicates that RD1-encoded early antigens, particularly ESAT-6 and CFP-10, are highly specific for the *Mycobacterium tuberculosis* complex and demonstrate strong immunogenicity, forming a robust biological basis for modern IGRAs and recombinant antigen-based skin tests. ESAT-6/CFP-10-based IGRAs such as QuantiFERON-TB Gold Plus and T-SPOT.TB generally show high specificity and acceptable sensitivity across diverse adult populations and outperform the TST in BCG-vaccinated settings. However, reduced sensitivity and higher rates of indeterminate results are consistently reported among children aged under five years and immunocompromised individuals.

ESAT-6/CFP-10-based skin tests, including C-Tb, ECT formulations and analogues, demonstrate diagnostic accuracy broadly comparable to IGRAs while clearly exceeding that of TST in BCG-vaccinated populations. These recombinant skin tests may offer operational advantages in settings where laboratory infrastructure is limited. Latency-associated antigens regulated by the DosR dormancy regulon and resuscitation-promoting factors (Rpf) appear to add incremental discriminatory information when combined with RD1 antigens, although further standardization and large-scale validation are still required.

Early-antigen-induced biomarkers, particularly IP-10 and multi-analyte cytokine signatures, show promise for improving diagnostic performance and potentially supporting risk stratification. Nevertheless, most biomarker-based approaches remain primarily within the research domain, and their prognostic value has not yet been established for routine clinical application.

### 3.8. Clinical Implications

The use of early *Mycobacterium tuberculosis* antigens, particularly ESAT-6 and CFP-10 encoded within the RD1 region, has important clinical implications for screening LTBI. Antigen-specific interferon-gamma release assays and ESAT-6/CFP-10-based skin tests provide higher specificity than the tuberculin skin test in BCG-vaccinated populations, enabling more accurate identification of individuals truly infected with *M. tuberculosis* and supporting targeted preventive therapy. These tools are especially valuable for healthcare workers, close contacts of infectious TB cases, migrants from high-burden settings, and persons scheduled to receive immunosuppressive treatment. ESAT-6/CFP-10-based skin tests represent a feasible alternative where laboratory capacity is limited. However, currently available antigen-based assays cannot reliably distinguish LTBI from active TB or predict progression to disease. Therefore, diagnostic results should be interpreted together with clinical risk assessment and guideline-based recommendations, particularly in high-risk and immunocompromised populations [35,36,37,38,39,40].

## 4. Discussion

This review synthesizes current evidence indicating that early *Mycobacterium tuberculosis* antigens, particularly the RD1-encoded proteins ESAT-6 and CFP-10, constitute the immunological backbone of contemporary tools for the detection of tuberculosis infection [10,11,12,13,14]. Their absence from Mycobacterium bovis BCG strains and most non-tuberculous mycobacteria explains the superior specificity of antigen-based assays compared with the tuberculin skin test, especially in BCG-vaccinated populations [3,7,10,11,12,13,14]. Rather than reiterating diagnostic accuracy metrics, the present discussion focuses on the biological meaning, clinical interpretation, and future implications of early-antigen-based immune readouts.

A fundamental limitation emerging from the analyzed studies is that ESAT-6/CFP-10-based interferon-gamma release assays and recombinant skin tests measure antigen-specific T-cell memory and immune sensitization, but do not define the biological state of infection [3,6,17,18,30,31,32]. A positive response reflects prior or ongoing exposure to *M. tuberculosis* antigens, yet cannot distinguish latent infection from incipient, subclinical, or early active disease [6,30,31,32]. This conceptual distinction is particularly important in high-risk groups, such as close contacts and immunocompromised individuals, in whom progression to active tuberculosis is more frequent and microbiological confirmation remains essential [17,18,19,20,34].

The introduction of CD8^+^-targeting antigen tubes in newer IGRA platforms has provided insights into the kinetics of antigen-specific cellular immunity, with several studies suggesting that CD8-dominant responses may be associated with recent or more intense antigenic stimulation [16,18,19,20]. However, the clinical utility of these quantitative or phenotypic differences for stage discrimination or individual risk stratification remains limited [18,30,31]. Similarly, serial testing in occupational cohorts reveals substantial conversion–reversion dynamics around assay cut-offs, indicating that fluctuations in RD1-specific T-cell responses often reflect biological and analytical variability rather than true changes in mycobacterial burden [18,19,34].

Latency-associated antigens regulated by the DosR dormancy regulon and resuscitation-promoting factors represent an important conceptual extension beyond RD1 proteins. Preferential recognition of these antigens in individuals with LTBI compared with active disease supports the hypothesis that immune targeting of metabolic states characteristic of non-replicating persistence may provide complementary information to early secretory antigens [27,28,29,30,31]. Nevertheless, heterogeneity in antigen selection, assay platforms, and outcome definitions, together with the lack of large-scale longitudinal validation, currently precludes their translation into routine diagnostics [29,30,31,36,37,38].

Host-derived biomarkers induced by early antigens, particularly IP-10 and multi-cytokine signatures, further illustrate the potential to move beyond single-analyte IFN-γ readouts [29,30,31,32,33]. While such approaches may improve sensitivity in children and immunocompromised patients and enable simplified sampling formats, their true added value lies in the possibility of developing prognostic rather than purely diagnostic tools [30,31,32,33]. This need is explicitly articulated in the World Health Organization Target Product Profiles for tuberculosis diagnostics, which prioritize assays capable of identifying individuals at highest risk of progression from latent infection to active disease [4,7,34].

At present, neither ESAT-6/CFP-10-based IGRAs nor recombinant skin tests meet these prognostic performance targets. Higher quantitative responses and recent test conversions are associated with increased relative risk, yet absolute predictive value remains low [3,6,30,31,32,34]. Consequently, preventive therapy decisions continue to rely primarily on epidemiological and clinical risk stratification rather than biomarker-defined progression risk [6,34].

In summary, early *M. tuberculosis* antigens remain indispensable for specific detection of infection and for BCG-independent immunodiagnosis [3,7,10,11,12,13,14]. However, their greatest future impact may lie not in further refinement of binary infection testing, but in integration with latency-associated antigens, host–response signatures, and longitudinal modeling to achieve stage-resolved and prognostically informative diagnostics. Harmonization of assay interpretation, validation of extended antigen panels, and alignment with WHO-defined predictive targets represent critical steps toward a new generation of risk-stratified tools for tuberculosis prevention and control [4,7,34,37,38,39,40].

Limitations of the Review. This review was conducted as a narrative (non-systematic) synthesis of the literature and is therefore subject to several limitations. First, although major biomedical databases were searched, no registered protocol was followed and no formal risk-of-bias assessment tools were applied, which may introduce selection bias and limit reproducibility. Second, the included studies were heterogeneous with respect to design, participant characteristics, assay platforms, antigen panels, cut-off values and reference standards, precluding quantitative meta-analysis and limiting direct comparison of diagnostic performance across studies. Third, publication bias cannot be excluded, as studies with statistically significant or positive findings are more likely to be published. Fourth, evidence remains relatively limited for some priority populations, including children under five years of age, elderly persons and people living with HIV or other causes of severe immunosuppression, restricting the generalisability of conclusions to these groups. Finally, most available data focus on diagnostic rather than prognostic performance, meaning that the ability of early antigen-based assays to predict progression from latent infection to active tuberculosis remains insufficiently characterised. These limitations should be considered when interpreting the findings of this review.

## 5. Conclusions

Early RD1-encoded *Mycobacterium tuberculosis* antigens, particularly ESAT-6 and CFP-10, have established a new paradigm for BCG-independent immunodiagnosis of tuberculosis infection and have enabled highly specific identification of infected individuals across diverse epidemiological settings [3,7,10,11,12,13,14]. Beyond their diagnostic utility, accumulating evidence indicates that the biological information captured by early and latency-associated antigen-specific immune responses reflect distinct phases of host–pathogen interaction, ranging from recent exposure to non-replicating persistence [27,28,29,30,31].

Despite these advances, current antigen-based assays remain limited to the detection of immune sensitization and do not yet provide reliable discrimination between latent, incipient, and active tuberculosis, nor do they accurately predict progression to disease [3,6,30,31,32,34]. This gap, explicitly recognized in the World Health Organization Target Product Profiles, underscores the need for a shift from purely binary infection tests toward prognostic and stage-resolving tools [6,34].

Future progress will depend on the rational integration of RD1 antigens with dormancy- and resuscitation-associated proteins, together with host–response biomarkers and quantitative immune signatures, validated in large longitudinal cohorts [29,30,31,32,33,36,37,38]. Harmonization of assay platforms, standardization of interpretation, and development of robust predictive models will be essential to translate these advances into risk-stratified preventive strategies and to strengthen global efforts toward tuberculosis elimination [6,29,30,31,32,33,34].

## Figures and Tables

**Table 1:** Immunological characteristics of early and latency-associated *Mycobacterium tuberculosis* antigens relevant for LTBI immunodiagnosis.

Antigen	Gene/Locus	Expression Stage	Immune Recognition	Functional Role	Diagnostic Relevance	Key References
ESAT-6	esxA (RD1)	Early secreted	CD4^+^, CD8^+^ Th1 (IFN-γ, TNF-α, IL-2)	ESX-1 secretion, membrane disruption, granuloma modulation	Core antigen in IGRAs and ESAT-6/CFP-10 skin tests; high specificity for MTBC	[10,11,12,13,14,15]
CFP-10	esxB (RD1)	Early secreted	CD4^+^, CD8^+^ Th1	ESX-1 complex formation, virulence	Paired with ESAT-6 in all commercial IGRAs and recombinant skin tests	[10,11,12,13,14,15]
TB7.7	Rv2654c (RD1-associated)	Early secreted	CD4^+^ T-cell IFN-γ	Accessory RD1 antigen	Modest sensitivity gains in some IGRAs, esp. in PLHIV	[16,17,18]
Rv1733c	DosR regulon	Dormancy	Polyfunctional CD4^+^ T cells	Hypoxic persistence	Preferentially recognized in LTBI vs. active TB	[27,28,29,30]
Rv2626c	DosR regulon	Dormancy	CD4^+^ Th1	Metabolic adaptation	Potential LTBI marker in multi-antigen panels	[27,28,29,30,31]
RpfA-E	rpf genes	Reactivation	Mixed CD4^+^/CD8^+^	Resuscitation from dormancy	Complementary to RD1 in discriminating latent vs. incipient TB	[29,30,31]

**Table 2:** Diagnostic performance of ESAT-6/CFP-10-based interferon-gamma release assays in LTBI.

Population	Test (Platform)	Reference Standard	Sensitivity (Range)	Specificity (Range)	Indeterminate Rate	Main Observations	Key References
Adults (general population)	QFT-Plus	Active TB/exposure	~80–90%	>95%	Low	High specificity in BCG-vaccinated settings; TB1/TB2 tubes capture CD4^+^ and CD8^+^ responses	[15,16,17,18,19]
Recent contacts	QFT-Plus	Exposure history	~85–90%	>95%	Low-moderate	TB2 (CD8) responses more frequent in recent infection and incipient TB	[16,18,19,20]
Adults (general population)	T-SPOT.TB	Active TB/exposure	~80–90%	>95%	Low	Comparable accuracy to QFT-Plus; standardized PBMC input	[15,16,17,21]
Healthcare workers (serial testing)	QFT-Plus, T-SPOT.TB	Serial conversion	Variable	High	Low-moderate	Frequent conversions/reversions near cut-off; quantitative changes more informative than dichotomous results	[18,19,34]
Children (>5 years)	QFT-Plus, T-SPOT.TB	Contact tracing	~75–90%	>95%	Moderate	Good concordance with TST, higher specificity in BCG-vaccinated children	[21,22,23,24,25,26]
Young children (<5 years)	QFT-Plus, T-SPOT.TB	Contact tracing	Reduced	High	Higher	Lower sensitivity and more indeterminate results due to immune immaturity	[21,22,23,26]
People living with HIV	QFT-Plus, T-SPOT.TB	Clinical diagnosis	~60–85%	High	Increased	Reduced sensitivity and higher indeterminate rates; TB2 responses may add value	[16,17,18,19,20,29,34]
Other immunocompromised (transplant, CKD, autoimmune)	QFT-Plus, T-SPOT.TB	Clinical diagnosis	Variable	High	Increased	Impaired T-cell responses; dual testing strategies sometimes used	[17,18,19,20,34]

**Table 3:** ESAT-6/CFP-10-Based Skin Tests for the Immunodiagnosis of LTBI.

Test	Antigen Composition	Population Studied	Reference Standard	Sensitivity (Range)	Specificity (Range)	Optimal Cut-Off	Safety Profile	Key Findings	Key References
C-Tb	Recombinant ESAT-6/CFP-10 fusion protein	Adults, children, PLHIV	Culture-confirmed TB, contact status	~75–85%	>95% in BCG-vaccinated	≥5–8 mm	Mild local reactions	Diagnostic accuracy comparable to IGRAs; higher specificity than TST in BCG-vaccinated settings	[21,22,23]
ECT (China)	ESAT-6/CFP-10 recombinant proteins	Adults, children	Culture-confirmed TB, exposure	~75–90%	>95%	≥5 mm	Mild local reactions	Higher specificity than TST; sensitivity similar to IGRAs	[24,25,26,27]
Diaskintest	ESAT-6/CFP-10 recombinant fusion	Adults, pediatric cohorts	Active TB, LTBI screening	~80–90%	>95%	≥5 mm	Mild local reactions	High concordance with IGRAs; superior specificity vs. TST	[28,29,30]
C-Tb in PLHIV	ESAT-6/CFP-10 fusion	HIV-infected adults	Clinical TB diagnosis	~65–80%	>95%	≥5 mm	Comparable to TST	Retains acceptable sensitivity and high specificity in immunocompromised	[22,23]

**Table 4:** Latency-Associated Antigens and Host Biomarkers Relevant for LTBI Immunodiagnosis and Risk Stratification.

Marker/Antigen Group	Representative Targets	Expression Stage	Immune Readout	Discrimination Potential	Added Value vs. RD1 Antigens	Limitations	Key References
DosR regulon antigens	Rv1733c, Rv2626c, Rv2628, Rv2004c	Dormancy/non-replicating persistence	CD4^+^ Th1 IFN-γ, IL-2, polyfunctional T cells	Higher responses in LTBI than in active TB	Complementary to ESAT-6/CFP-10; may improve stage discrimination	Heterogeneous panels; lack of standardization	[27,28,29,30,31]
Resuscitation-promoting factors (Rpf)	RpfA-E	Reactivation/metabolic transition	CD4^+^ and CD8^+^ IFN-γ responses	Limited alone; supportive in combined panels	Add information on reactivation biology	Low standalone diagnostic accuracy	[29,30,31]
IP-10 (CXCL10)	IFN-γ-inducible chemokine	Host response to RD1 stimulation	Plasma/supernatant IP-10	Sensitivity ≥ IFN-γ in some groups	Improves detection in children, PLHIV, immunosuppressed	Slight loss of specificity in dual-marker algorithms	[29,30,31,32,33]
Multi-cytokine signatures	IFN-γ, IP-10, IL-2, TNF-α, GM-CSF	Host immune profile	Multiplex cytokine patterns	Improved separation of LTBI vs. active TB in models	Potential prognostic stratification	Complex analytics; no large prospective validation	[30,31,32,33]
Machine-learning classifiers	Integrated antigen + cytokine panels	Systems immunology	Multivariate immune signatures	Experimental prediction of disease states	Conceptual step toward prognostic tests	Not standardized; research use only	[30,31,32,33,36,37,38]

## Data Availability

No new data were created or analyzed in this study. Data sharing is not applicable to this article.

## Abbreviations

The following abbreviations are used in this manuscript:

BCG	Bacille Calmette–Guérin
CFP-10	Culture Filtrate Protein-10
DosR	Dormancy Survival Regulator
ECT	ESAT-6/CFP-10 Skin Test
ELISA	Enzyme-Linked Immunosorbent Assay
ESAT-6	Early Secreted Antigenic Target-6
HIV	Human Immunodeficiency Virus
IFN-γ	Interferon-Gamma
IGRA	Interferon-Gamma Release Assay
IL-2	Interleukin-2
IP-10 (CXCL10)	Interferon-Gamma-Induced Protein-10
LTBI	Latent Tuberculosis Infection
*M. tuberculosis*	*Mycobacterium tuberculosis*
NTM	Nontuberculous Mycobacteria
PBMC	Peripheral Blood Mononuclear Cells
PPD	Purified Protein Derivative
QFT-Plus	QuantiFERON-TB Gold Plus
RD1	Region of Difference-1
Rpf	Resuscitation-Promoting Factor
TB	Tuberculosis
TST	Tuberculin Skin Test
TNF-α	Tumor Necrosis Factor-Alpha
